# Defining Safe Light Intensity Limits of Near‐Infrared Illumination Avoiding Skin Heating in Medical Optical Diagnostic Methods

**DOI:** 10.1002/jbio.70320

**Published:** 2026-06-30

**Authors:** Anna‐Lena Sahlberg, Sophie Helene von der Sahle, Sara Bergsten, Henrik Palme, Emilie Krite Svanberg

**Affiliations:** ^1^ Department of Physics Lund University Lund Sweden; ^2^ Lund Laser Centre, Lund University Lund Sweden; ^3^ Neola Medical AB Lund Sweden; ^4^ Department of Biomedical Engineering and Health Systems KTH Royal Institute of Technology Stockholm Sweden; ^5^ Department of Clinical Sciences, Division of Pediatric Anesthesiology and Intensive Care Skåne University Hospital Lund Sweden

**Keywords:** dermal probe, diode laser, free beam, in vivo, laser safety, optical spectroscopy, skin tone, tissue temperature increase

## Abstract

This study examined the thermal response of human skin in vivo to near‐infrared illumination (764 nm) delivered either as a free laser beam or through direct contact using a dermal diffusive probe. The aim was to quantify skin heating under increasing illumination intensities and to identify safe exposure thresholds for different skin tones and illumination methods. Research participants with skin tones 1–5 (ranging from light to brown) were included. The results show that skin tone significantly influenced heating under free‐beam illumination, whereas temperature increases with dermal probe illumination showed no significant dependence on skin tone. The maximum safe irradiance was determined to be 7.6 mW/mm^2^ for free‐beam illumination and 2.3 mW/mm^2^ for dermal probe illumination, corresponding to a maximum skin temperature of 43°C. These limits apply to skin tones 1–5, and further research is required to establish safety thresholds for skin tone 6.

## Graphical abstract

The study measured skin heating from 764 nm illumination using free‐beam and dermal‐probe methods across a range of skin tones. Free‐beam heating rose with darker tones, while probe contact increased temperature independent of skin tone. Estimated safe exposure limits support design of future optical medical diagnostic devices.

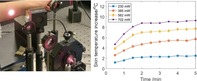

**Abbreviations**
GASMAS: gas in scattering media absorption spectroscopy
LED: light‐emitting diode
NIR: near‐infrared

## 1 Introduction

Optical spectroscopy in the near‐infrared (NIR) wavelength region forms the basis of numerous medical diagnostic and monitoring technologies. This spectral region, often referred to as the “tissue optical window,” allows light to penetrate up to several centimeters into tissue (see e.g., [1]). Visible light is more strongly absorbed by hemoglobin, limiting the penetration depth, while wavelengths longer than approximately 1 μm are more heavily absorbed by water. Melanin absorption at the skin surface also affects light penetration, with darker skin contributing to reducing penetration compared to lighter skin [2, 3]. Consequently, many optical techniques for medical diagnostics employ NIR wavelengths, where absorption by hemoglobin, melanin and water is reduced. Representative examples include pulse oximetry, which operates at 660–1100 nm with powers of ~4 to 10 mW [4]; Raman spectroscopy, which uses ~785 nm excitation at ~10 to 300 mW for in vivo tissue characterization [5, 6, 7]; and Optical Coherence Tomography (OCT), used for the diagnosis of skin disorders, which typically operates within the 900–1300 nm range at a few milliwatts [8], although some systems employ up to 20 mW at 800 nm [9].

GAs in Scattering Media Absorption Spectroscopy (GASMAS) is a technique developed to detect free gas molecules in cavities embedded within scattering and absorbing materials [1]. A promising medical application of GASMAS is continuous lung monitoring in infants, enabling early detection of respiratory complications [10, 11, 12, 13, 14]. This approach employs 760–764 nm light to target the strongest oxygen gas absorption lines in the NIR, with typical application powers of about 10–30 mW. As tissue absorption and scattering limit penetration depth to a few centimeters, current clinical application is limited to preterm and term infants. However, preliminary results from porcine lung models show promise for extending the method to older infants and children [15]. Achieving this may require increasing the optical power to several hundred milliwatts, which raises concerns about possible thermal tissue damage. Ensuring safe application of GASMAS, and other medical laser techniques using similar wavelengths, therefore requires careful evaluation of exposure limits to prevent thermal damage to the skin and underlying tissue.

According to the American National Standards Institute (ANSI Z136.3), the maximum permissible skin exposure for continuous illumination in the 700–1050 nm range is 0.30 W/cm^2^ [16]. This limit is intentionally conservative and generally not derived from systematic in vivo experimental validation at specific wavelengths. Photothermal effects of NIR laser irradiation on human skin have, to the best of our knowledge, been investigated primarily through computational or ex vivo studies at specific wavelengths (see, e.g., [17, 18, 19]). However, light‐tissue interactions are highly complex, and the optical absorption properties governing the energy deposition are strongly wavelength‐dependent. Also, while tissue heating can be estimated using light‐tissue interaction models, these often neglect heat dissipation mechanisms such as convection, evaporation, and thermal radiation. In addition, physiological factors such as blood perfusion dynamically regulate tissue temperature, further complicating accurate prediction of thermal effects. As a result, establishing precise, wavelength‐specific safe exposure limits from existing data remains challenging. In particular, experimental in vivo data at wavelengths around 760–764 nm are lacking.

To our knowledge, the present study represents the first systematic in vivo investigation of safe intensity limits for 764 nm illumination on human skin. The study examines both the thermal response and temporal dynamics of skin heating under NIR illumination delivered either as a free laser beam or via direct contact using a dermal diffusive probe. The aim is to quantify temperature increases under increasing illumination intensities and to identify safe exposure thresholds across different skin tones and illumination methods. This research is essential for enabling the safe clinical application of optical diagnostic techniques operating near 764 nm, particularly in applications such as GASMAS, where higher optical powers may be required.

## 2 Experimental Section

### 2.1 Experimental Setup

The laser system used in this study was a tapered amplified diode laser system (Toptica Photonics DLC TA pro 765) equipped with a fiber coupler. The system consisted of an external cavity diode laser (764 nm) amplified to an output power of up to approximately 800 mW.

**FIGURE 1:**
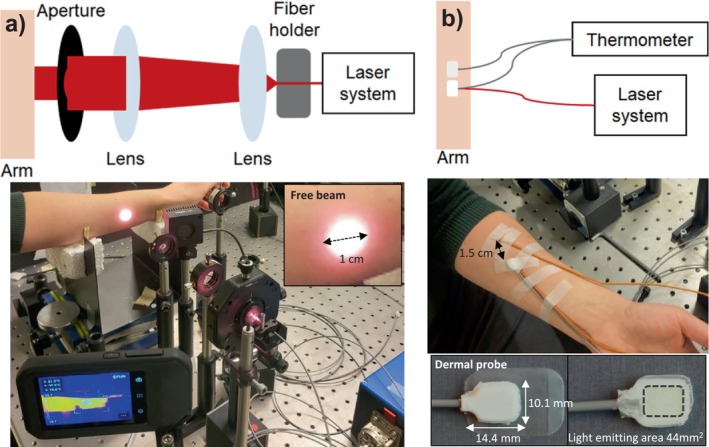
Schematic and photographs of the setup for (a) free‐beam and (b) dermal probe measurement.

For the free‐beam measurements, the light from the TA pro was delivered through a multimode optical fiber. The distal end of the fiber was mounted and three convex lenses were used to collimate the light and achieve the desired beam size. The laser light was aligned to illuminate a spot on the middle of the inner forearm, see Figure 1a. The laser beam had an approximately top‐hat profile with a beam diameter adjusted to 1.0 cm using an aperture, resulting in an illumination area of 79 mm^2^. The position was marked to ensure consistency throughout the measurements for each research participant. Illumination was applied for 5 min, and the skin temperature in the illuminated spot was monitored using an infrared camera (FLIR C5). The temperature was recorded at the illuminated spot and at a reference point located 1.5 cm away.

For the dermal probe measurements, the light from the TA pro laser was delivered through a dermal emitter probe, consisting of an optical fiber attached to a diffusing probe head with an illuminating area of 44 mm^2^, see Figure 1b. The probe was secured in direct contact with the skin using surgical tape. The skin temperature was monitored with two thermocouples (Fluke 52II thermometer, 80PK‐1 probe): one placed between the dermal probe and the skin, and the other positioned 1.5 cm away for reference measurements. The infrared camera and the thermocouples recorded similar temperatures to within ±2°C of each other and exhibited similar response patterns.

For both configurations, the skin temperature increase was recorded at four different illumination power levels, starting from the lowest. The laser power was adjusted using the fiber coupler and verified with a thermal power meter. The free‐beam measurements employed output powers between 200 and 750 mW, while the dermal probe measurements used lower power levels between 20 and 120 mW. The power was measured immediately before and after illumination to ensure a stable power level during the measurement, and < 10% power variation was observed during the experiments.

Medical devices must comply with IEC 60601‐1 Medical Electrical Equipment Safety standards [20], which specify temperature limits for any part of an instrument in contact with patients. For devices not intended to heat tissue, the temperature should not exceed 41°C, although temperatures up to 43°C are permitted if justified by risk management [20]. Some exceptions exist; for example, transcutaneous PCO_2_ measurements in neonatal infants involve heating up to 44°C [21, 22]. To comply with existing regulations, the current study set a safety limit of a maximum skin temperature of 43°C during exposure to the light.

The measurement procedure was as follows: a baseline skin temperature was recorded, followed by initiation of laser illumination. The skin temperature was measured every 30 s for 5 min. After each measurement, a cool‐down period of at least 2 min was observed before the next measurement. If the skin temperature reached 43°C, the measurement was terminated and no further power was applied. The cool‐down period was not constant, as adjustments to the laser power sometimes required additional time, particularly for the dermal probe measurements where reattachment was necessary. The cooling time also varied among participants, depending on how long the skin required to return to the baseline temperature. However, a minimum of 2 min was maintained for all cases.

### 2.2 Clinical Protocol

Ethical approval for the study was obtained from the Swedish Ethical Review Authority (reference number 2024‐02783‐01). Inclusion criteria required research participants to be over 18 years of age and in good health. Participants were also required not to be pregnant and to have no recent visible sun exposure or any skin conditions (e.g., rosacea, eczema, skin infections, or excessive scarring or keloid formations) that could affect the thermal response of the skin to NIR light. Additionally, research participants with varying skin tones were included to ensure that the findings are representative and generalizable to patients with diverse ethnic and genetic backgrounds. Healthy volunteers were recruited through informational flyers describing the purpose and procedures of the study.

After providing informed consent, research participants completed a questionnaire confirming compliance with the inclusion criteria. The protocol recorded parameters potentially correlated with skin heating, such as age, weight (related to subcutaneous fat) and gender. Skin tone was classified using a colorimeter (Skin Color Catch, Delfin Technologies) which measures the skin reflectance and calculates the color parameters according to the CIELAB color space. The device calculates the Individual Typology Angle (ITA°) based on the luminosity (L*) and yellow value (b*), to provide an objective skin tone classification [23, 24]. ITA° values are correlated to melanin content and categorize skin into six different skin tones, as shown in Table 1. Measurements were taken directly at the skin area designated for laser illumination.

**TABLE 1:** Skin tone classification according to the ITA° [23].

Skin tone	Skin tone classification	ITA° range
1	Very light	55–90
2	Light	41–54
3	Intermediate	28–40
4	Tanned	10–27
5	Brown	−30 to 9
6	Dark	−90 to −29

To estimate subcutaneous fat thickness, a digital caliper was used to measure a skinfold on the forearm. Three consecutive measurements were performed, and the average value was used for analysis. This measurement was conducted after temperature recordings to avoid any irritation from pinching the skin, which could interfere with the thermal characterization. Table 2 summarizes research participant data collected during the protocol. Each session, including consent, parameter collection, and measurements, lasted approximately 90 min, with the measurement procedure comprising the majority of the time.

**TABLE 2:** Parameters recorded during the measurement procedure.

Questionnaire parameters	Measured parameters
Age	*Before the procedure*:
Weight	ITA°
Gender (M/F)	
Pregnant? (Y/N)	*During the procedure*:
Skin conditions?	Skin temperature and laser power
Ongoing medication?	
Recent sunburn?	*After the procedure*:
History of keloid formation?	Skinfold thickness (subcutaneous fat)

### 2.3 Data Evaluation Methods

The study employed a repeated‐measures design, with the skin temperature recorded during incremental increases in light power. The primary objective was to determine how tissue heating depends on illumination intensity and its correlation with skin tone. The heating was assessed for both free‐beam and dermal probe illumination. The goal was to establish maximum permissible intensity levels for optical diagnostic applications using 764 nm illumination of the skin. Statistical significance was evaluated at a threshold of *p* < 0.05.

The sample size was set at five research participants per skin tone, as outlined in Table 1. This was determined to be sufficient based on a power calculation for a one‐way repeated‐measures analysis of variance (ANOVA) using sample data. Skin heating parameters were analyzed using one‐way ANOVA to assess differences among skin tones, and all data analysis was performed using MATLAB (MathWorks Inc. R2022a). When ANOVA indicated a significant difference (*p* < 0.05), post hoc Tukey's multiple comparison tests were conducted to identify which skin tone groups differed significantly from each other.

## 3 Results and Discussion

### 3.1 Research Participants

A total of 25 research participants with skin tones ranging from 1 to 5 took part in the study, with five participants in each group. Recruitment of research participants with skin tone 6 proved challenging in the current setting; therefore, this group was not included. The average age of the research participants was 36.5 ± 12.5 years, the average weight was 77.1 ± 12.4 kg, and 11 of the 25 subjects were female.

### 3.2 Baseline Skin Temperature

**FIGURE 2:**
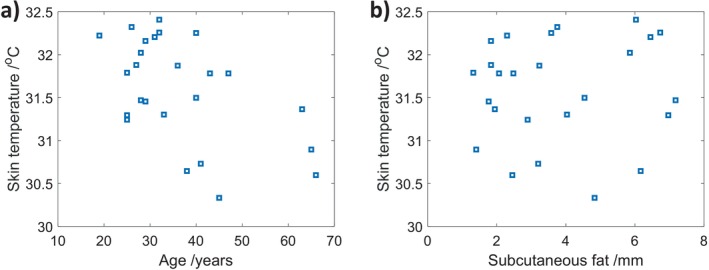
Baseline skin temperature versus (a) participant age and (b) subcutaneous fat thickness measured using the skinfold technique.

Initial skin temperature showed no correlation with gender, averaging 31.6°C ± 0.7°C for females and 31.6°C ± 0.6°C for males. Figure 2 illustrates skin temperature versus participant age and subcutaneous fat thickness measured using the skinfold technique. A moderate negative correlation was observed between skin temperature and age (*r* = −0.53, *p* = 0.0067), indicating that younger research participants generally exhibited slightly higher skin temperatures, although individual variability was substantial. This finding aligns with a review reporting that older individuals typically have lower resting skin temperatures [25]. No significant correlation was found between skin temperature and subcutaneous fat thickness, although previous studies suggest that higher fat content can reduce skin surface temperature [26]. Given the limited accuracy of the skinfold method, measurement errors may have obscured any correlation. Future studies could employ more precise techniques, such as ultrasound, to assess subcutaneous fat and its potential influence on skin temperature.

### 3.3 Skin Temperature Elevation and Demographic Factors

The primary aim was to assess how skin heating depends on illumination intensity using either a free laser beam or a dermal probe, and whether heating correlates with skin tone. The sample size did not permit detection of significant differences related to gender, age, or subcutaneous fat thickness. Larger studies are needed to evaluate these factors.

### 3.4 Skin Temperature Elevation and Skin Tone

#### 3.4.1 Free‐Beam Measurement

**FIGURE 3:**
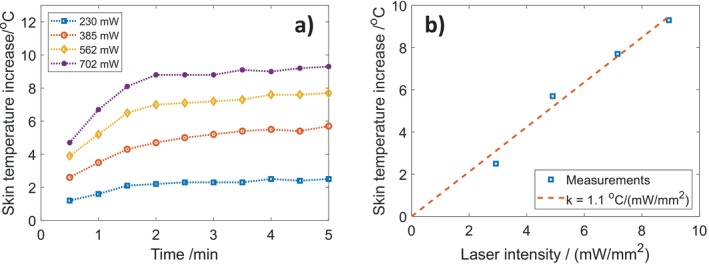
(a) Skin temperature increase under free‐beam illumination for different laser powers, measured on one research participant with skin tone 3. (b) Dependence of temperature increase on laser intensity, with the linear fit yielding a skin heating parameter of 1.1°C/(mW/mm^2^).

The skin temperature was recorded for four illumination intensities ranging from 200 to 750 mW over an area of 79 mm^2^, corresponding to 2.5–9.5 mW/mm^2^. Figure 3a shows the results from one research participant with skin tone 3, illustrating the skin temperature increase over 5 min for four laser power levels. The temperature stabilized after approximately 2–3 min. The temperature increase for each measurement was defined as the difference between the maximum recorded skin temperature and the initial baseline temperature.

Figure 3b presents the maximum skin temperature increase as a function of the laser intensity (power per mm^2^). A skin heating parameter was defined as the slope of the linear relationship between temperature increase and laser intensity, which for this series was 1.1°C/(mW/mm^2^). The increase in temperature demonstrated a statistically significant dependence on the illumination intensity, confirming that skin heating at 764 nm increases with higher laser intensity.

**FIGURE 4:**
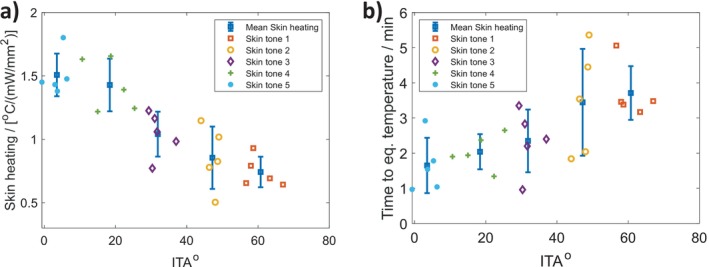
(a) Skin heating parameter for the free‐beam measurements versus the ITA°, grouped by skin tone. (b) Time required to reach stable temperature for all research participants versus ITA°.

Figure 4a shows the skin heating parameter for the free‐beam measurements for all research participants versus ITA°, grouped by skin tone (1–5). The parameter indicates the temperature increase in °C per 1 mW/mm^2^ of illumination. Error bars represent mean values and standard deviation within each skin tone group. The skin heating generally increased with decreasing ITA° (i.e., darker skin tones), although variability within groups was substantial. For example, the lowest skin heating was observed in skin tone 2, while one research participant with skin tone 3 exhibited lower skin heating than several skin tone 1 or 2 participants. ANOVA revealed no significant differences among skin tones 1–3, or between skin tones 4–5. However, heating for skin tones 4 and 5 was significantly greater than for skin tones 1–3. These findings indicate a statistically significant difference in heating between lighter (1–3) and darker skin tones (4–5).

Figure 4b shows the time required to reach a stable skin temperature, determined by fitting an exponential saturation model and defining stability as 95% of the asymptotic value. Darker skin tones tended to heat faster, while lighter skin tones required longer to reach equilibrium temperature. The only statistically significant difference was between skin tone 1 (average 3 min 43 s) and skin tone 5 (average 1 min 39 s). For two participants with lighter skin tones, the fitted model yielded asymptotic times extrapolated beyond the 5 min measurement window. This suggests that, in these cases, a steady‐state temperature may not have been fully reached during the illumination period, although some uncertainty in the model fit remains due to the limited number of data points. The majority of measurements reached a stable temperature within 5 min, supporting the assumption that the recorded skin temperatures increases in each case are representative of steady‐state conditions.

#### 3.4.2 Dermal Probe Measurement

**FIGURE 5:**
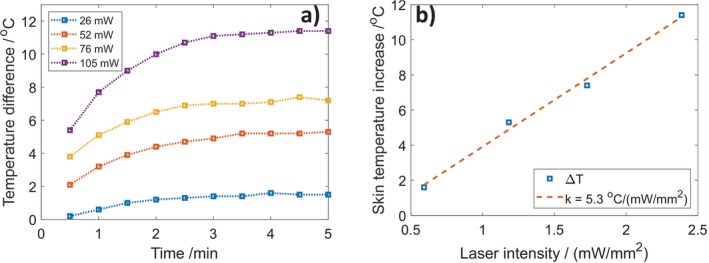
(a) Skin temperature increase under dermal probe illumination for different laser powers, measured on one research participant with skin tone 3. (b) Dependence of temperature increase on laser intensity, with the linear fit yielding a skin heating parameter of 5.3°C/(mW/mm^2^).

The skin temperature during dermal probe illumination was recorded for four different power levels ranging from 20 to 110 mW over an area of 44 mm^2^, corresponding to 0.5–2.5 mW/mm^2^. Figure 5a shows data from a research participant with skin tone 3, illustrating the skin temperature increase over 5 min for four laser power levels. Figure 5b presents the maximum temperature increase as a function of the laser intensity. The temperature plateau was reached after approximately 4–5 min. For this measurement series, the skin heating parameter was 5.3°C/(mW/mm^2^), with a statistically significant dependence on the illumination intensity.

**FIGURE 6:**
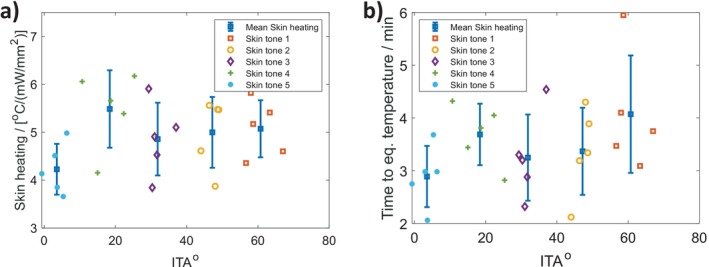
Skin heating parameter for the dermal probe measurements versus ITA°, grouped by skin tone. (b) Time required to reach stable temperature for all research participants versus ITA°.

Figure 6a shows the skin heating parameter for the dermal probe measurements for all research participants versus ITA°, grouped by skin tone (1–5). Comparison of Figures 4a and 6 a indicates that the dermal probe illumination produced significantly greater heating than the free‐beam illumination. Individual measurements are shown as dots, and error bars represent mean values and standard deviation within each skin tone group. Unlike the free‐beam measurements, dermal probe heating did not differ significantly among skin tones.

Figure 6b shows the time required to reach stable temperature for all research participants as a function of ITA° and skin tone. No statistically significant dependence on skin tone was observed for the dermal probe measurements. However, comparison of free‐beam and dermal probe results revealed a significant difference in stabilization time: dermal probe measurements required, on average, 48 s longer to reach equilibrium. As in Figure 4b, there is one case where the fitted model yielded a steady‐state time extrapolated beyond the 5 min measurement window. Nevertheless, as the majority of measurements reached a stable temperature within 5 min, the recorded skin temperature increases are considered representative of steady‐state conditions.

#### 3.4.3 Sources of Measurement Error

One important source of error in the free‐beam setup is the consistency of the measurement location on the forearm. Although efforts were made to standardize positioning, it is uncertain whether illumination occurred at exactly the same spot during each trial for every research participant. Minor deviations in location could introduce variability in recorded temperature, given the natural heterogeneity of skin properties across even small surface areas.

Additionally, the infrared camera used for the free‐beam measurements has inherent limitations in accuracy and precision. With a specified accuracy of ±3°C and precision of ±0.07°C, the device can detect small temperature changes but may report absolute values offset from true skin temperatures. This could lead to discrepancies when comparing data across skin tones or individuals, particularly when temperature differences are subtle.

The dermal probe setup introduces a different set of potential errors due to its physical interaction with the skin. One major factor is the consistency of contact pressure between dermal probe, thermocouple, and skin. Variations in attachment tightness can affect thermal conduction, influencing the measured temperature rise. Loose contact may underestimate temperature increases, while overly tight contact could restrict blood flow or compress the skin, altering its thermal response. Another source of variability is the exact placement of the thermocouple between the probe and the skin. Small positional shifts can significantly affect the readings, especially if the thermocouple is not in optimal thermal contact with the skin or is misaligned relative to the dermal probe. In principle, the thermocouple both blocks and absorbs a portion of the light emitted by the dermal probe, which may influence the resulting skin temperature during illumination. However, given its small size relative to the illuminated area, this effect is likely negligible.

Furthermore, the location of the probe on the forearm relative to superficial blood vessels may introduce variability in skin cooling. Areas with higher vascular density could dissipate heat more rapidly due to increased blood flow, resulting in lower observed temperature increase. This physiological variability adds complexity to interpreting the dermal probe measurements. While similar effects may influence the free‐beam measurements, they are likely less pronounced because cooling in that setup is more strongly affected by evaporation and convection.

### 3.5 Discussion of Safe Light Powers for Continuous Optical Diagnostic Methods

The results demonstrate that skin heating under 764 nm laser illumination is strongly dependent on laser intensity. Table 3 summarizes the skin heating parameters for free‐beam and dermal probe measurements. For free‐beam illumination, heating was significantly influenced by skin tone, with skin tones 4–5 exhibiting greater temperature increases than skin tones 1–3. This effect is likely related to differences in optical properties, as darker skin absorbs more light due to higher concentrations of melanin [3, 27, 28], resulting in greater energy deposition at the same illumination intensity and consequently higher temperature increases. Darker skin tones also reached thermal equilibrium more rapidly, consistent with stronger initial absorption leading to faster heat accumulation and a shorter time to reach a steady‐state temperature.

**TABLE 3:** Skin heating parameter for free‐beam and dermal probe measurements. Free‐beam measurements showed significant differences among skin tones.

	Skin tone	Skin heating parameter [°C/(mW/mm^2^)]	Estimated temperature increase [°C] for fixed power and area
Free‐beam	1–3	0.88 ± 0.22	5.6°C [500 mW, 79 mm^2^]
	4–5	1.5 ± 0.18	9.5°C [500 mW, 79 mm^2^]
Dermal probe	1–5	4.9 ± 0.76	11°C [100 mW, 44 mm^2^]

In contrast, dermal probe illumination produced significantly higher overall skin heating compared to the free‐beam measurements, but showed no significant dependence on skin tones. Similarly, no differences in the time to reach thermal equilibrium were observed among participants with different skin tones in the dermal probe experiments.

The skin heating parameter can be used to estimate temperature increases for a given laser power and illumination area. Table 3 also provides examples of expected temperature increase for typical power settings: 500 mW for free‐beam illumination (79 mm^2^ area) and 100 mW for dermal probe (44 mm^2^ area). Based on these parameters, safe illumination limits for 764 nm light incident on the skin of the forearm can be estimated. Assuming an initial skin temperature of 31.6°C and a maximum permissible temperature of 43°C, free‐beam illumination over 79 mm^2^ should remain below 1000 mW for skin tones 1–3 and below 600 mW for skin tones 4–5. For dermal probe illumination over 44 mm^2^, power should not exceed 100 mW. It should be noted that this study did not include the full ITA° range for skin tone 5 (ITA° = −30 to 9). Research participants had ITA° ≥ −1; therefore, no conclusions can be drawn for individuals with ITA° < −1, which includes part of skin tone 5 and skin tone 6 (see Table 1). Given the observed trend of increased heating for darker skin tones in the free‐beam measurements, skin tone 6 may exhibit even higher heating, but further research is needed to confirm whether significant differences exist between skin tone 4–5 and skin tone 6.

Dermal probe illumination showed a significantly higher skin heating parameter, but no significant dependence on skin tone. This was unexpected, as both prior studies [2, 3] and the free‐beam results indicate that higher melanin content increases light absorption. A key difference between the two setups is heat dissipation: free‐beam measurements allow cooling through evaporation and convection, whereas the dermal probe inhibits these cooling mechanisms by physically blocking the skin surface, leading to higher skin temperatures. Although thermal radiation also contributes to cooling, it is typically negligible compared to the other mechanisms. During dermal probe illumination, heat dissipation relies primarily on tissue conduction and blood flow, which are less efficient than evaporative and convective cooling, leading to higher skin temperatures. As skin temperature increases, vasodilation increases dermal capillary blood flow, promoting heat transfer to circulating blood, a process affected by both probe placement and individual vascular anatomy. The dermal probe measurements also showed greater inter‐individual variability among participants with similar skin tones, suggesting differences in cooling efficiency independent of skin tone. While melanin is a key factor in optical absorption, skin thermal diffusivity is mainly determined by other physiological factors [29]. Variations in these factors may therefore mask melanin‐related differences, explaining the reduced skin‐tone dependence in the dermal probe measurements. Although similar mechanisms are present in the free‐beam measurements, convection and evaporation dominate heat transfer in that setup.

Additionally, because the dermal probe is in direct contact with the skin, light reflected from the skin surface may be partially re‐reflected by the probe, increasing the effective fluence rate of the illumination. This may also contribute to the higher skin heating observed in the dermal probe measurements. Since lighter skin reflects a larger fraction of incident 764 nm light than darker skin [2], this recapturing effect could result in relatively greater fluence enhancement for lighter skin tones, thereby reducing differences in absorbed energy between skin tones. Future studies could employ isotropic sensors to more accurately quantify fluence rate for the dermal probe measurements and its dependence on skin properties.

## 4 Conclusion

To our knowledge, this study represents the first systematic in vivo investigation of safe intensity limits for 764 nm illumination on human skin. The resulting safety thresholds are directly relevant for optical techniques relying on continuous‐wave illumination at this wavelength, such as GASMAS for lung monitoring. More broadly, these findings provide quantitative insight into photothermal effects in the NIR regime, although differences in optical absorption must be considered when extrapolating to other wavelengths. The temporal dynamics of skin heating observed in this study indicate that temperature increases are strongly time‐dependent during the first 30 s of illumination, suggesting that techniques employing shorter exposure times, such as OCT and Raman spectroscopy, may tolerate higher illumination powers than those identified under continuous exposure.

Both free‐beam and dermal probe configurations were examined in 25 research participants with skin tones 1–5. Skin tone was found to significantly influence skin heating under free‐beam illumination, with darker skin tones exhibiting greater temperature increases due to higher melanin content, whereas no such dependence was observed for dermal probe illumination. Based on these findings, maximum safe power levels for skin tones 1–5 were determined to be 600 mW for free‐beam illumination over an area of 79 mm^2^ and 100 mW for dermal probe illumination over 44 mm^2^, ensuring the skin temperature remains below the established safety threshold of 43°C [20]. However, as the study only included participants with skin tones down to an ITA° of −1, no definitive conclusions can be drawn for darker skin (part of skin tone 5 and all of skin tone 6). For lighter skin tones (1–3), free‐beam illumination up to 1000 mW over 79 mm^2^ appears safe under the investigated conditions.

Current diagnostic techniques commonly use low illumination powers to avoid thermal damage. However, future technological advancements involving higher optical powers to improve signal‐to‐noise ratios may increase the risk of thermal tissue damage. These results therefore provide an important basis for defining safe operating regimes and guiding the design of next‐generation optical diagnostic devices. Future work should include a larger and more diverse pool of research participants, particularly incorporating skin tone 6, to enable more comprehensive safety guidelines. Defining robust, wavelength‐specific exposure limits based on in vivo experiments is essential for the safe advancement of optical medical technologies and for their broader application in patient monitoring, diagnostics, and treatment across diverse patient populations.

## Author Contributions

Anna‐Lena Sahlberg is the lead author, with primary responsibility for the experimental design, final data analysis, and overall manuscript preparation, including figure generation and submission. Sophie Helene von der Sahle conducted the experiments, performed the initial data analysis, and contributed to writing the paper. Sara Bergsten provided guidance on experimental design and data analysis, and contributed to the writing and presentation of the results. Henrik Palme contributed substantially to the design of the experimental setup and measurement procedure, and contributed to the final edit of the paper. Emilie Krite Svanberg served as the principal medical advisor for the project, was responsible for obtaining ethical approvals and ensuring the safety of the experimental design, advised on data analysis and interpretation, and contributed to manuscript writing.

## Funding

Financial contributions for the project were received from Swelife and Medtech4Health through Vinnova [grant number: 2022‐03489].

## Conflicts of Interest

Sara Bergsten and Sophie Helene von der Sahle are employees of Neola Medical AB, a company that develops a lung monitoring device for preterm infants based on GASMAS technology using near infrared illumination. Sara Bergsten and Emilie Krite Svanberg are shareholders in Neola Medical AB. There are no direct financial interests in the outcome of this project.

## Data Availability

Research data are not shared.
